# Governing the moral economy: Animal engineering, ethics and the liberal government of science

**DOI:** 10.1016/j.socscimed.2012.02.049

**Published:** 2012-07

**Authors:** Alison Harvey, Brian Salter

**Affiliations:** a Centre for Biomedicine & Society, King's College London, Strand, London WC2R 2LS, UK; b Department of Political Economy, King's College London, Strand, London WC2B 4LL, UK

**Keywords:** Science governance, Governmentality, Liberal government, Cultural values, Bioethics, Human/animal mixing

## Abstract

The preferred Western model for science governance has come to involve attending to the perspectives of the public. In practice, however, this model has been criticised for failing to promote democracy along participatory lines. We argue that contemporary approaches to science policy making demonstrate less the failure of democracy and more the success of liberal modes of government in adapting to meet new governance challenges. Using a case study of recent UK policy debates on scientific work mixing human and animal biological material, we show first how a ‘moral economy’ is brought into being as a regulatory domain and second how this domain is governed to align cultural with scientific values. We suggest that it is through these practices that the state assures its aspirations for enhancing individual and collective prosperity through technological advance are met.

## Highlights

► We study UK policy debates on animal engineering: genetic modification and creation of interspecies embryos. ► We suggest that ethical issues in animal engineering are configured as part of a regulatory domain we term a ‘moral economy’. ► We show how the moral economy is made governable through neo-liberal practices that ensure support of science is maintained. ► We argue that this is not a failure of contemporary modes of science governance to enhance democracy. ► Rather, it demonstrates the success of liberal modes of government in adapting to meet new governance challenges.

## Introduction

Reflecting on science governance in its 2000 White Paper *Excellence and Opportunity: a science and innovation policy for the 21*st *century*, the Department of Trade and Industry observes that

Science is too important to be left only to scientists. Their knowledge, and their assessment of risks, is only one dimension of the challenge for society. When science raises profound ethical and social issues, the whole of society needs to take part in the debate. (DTI, 2000: 54)

The DTI was acknowledging that in the wake of very public controversies over science in the 1980s and 90s, including the response to genetically modified (GM) crops and the bovine spongiform encephalopathy (BSE) crisis, the preferred Western model for good science policy making could no longer rely exclusively on expert advisory committees but must include attendance to the perspectives of the public. This model has been given intellectual foundations by work in the sociology of science and normative credentials by the promotion of deliberative models of democracy. However, the manner in which the model has been put into practice in science governance has been criticised for neglecting the promotion of democracy along participatory lines. This paper argues that contemporary approaches to science governance demonstrate less the failure of democracy and more the success of liberal modes of government in adapting to meet new governance challenges.

A good deal of the work in science studies that has addressed the relations between scientific knowledge and political processes can be summed up as holding that “the technical is political, the political should be democratic, and the democratic should be participatory” (Moore, 2010a, 2010b: 793), leading to the criticism that science studies has taken its conception of politics ‘off the shelf’ (de Vries, 2007). More recently researchers have started to address this criticism through studies of the nature of the political in science governance (e.g. Braun, Herrmann, Könninger, & Moore, 2010; Braun, Moore, Herrmann, & Könninger, 2010; Brown, 2009; Thorpe, 2010; Thorpe & Gregory, 2010). Our paper contributes to this latter body of work. It has no normative intent; we do not present an argument as to how science policy should be made. Rather, we offer an analysis of how those who govern understand that policy should be made. Our account is restricted to examining the political rationalities underpinning science policy making and the practices and techniques advocated for effecting good government of this activity. We do not engage with the matter of what happens when these techniques and practice are put into operation – how they may meet resistance from those who are being governed, and are modified and re-shaped as other stakeholders become involved in the actual process of making and implementing policy decisions. Nevertheless, as Braun and colleagues have put it in a recent article that uses a similar Foucauldian-inspired approach to our own, an analysis of science policy making that ‘does not take the inclusion of “ethical concerns”, the recognition of plural viewpoints or practices of “engaging the public” *per se* as challenging established power relations’ can illuminate how these practices can have the effect of stabilizing prior commitments rather than exposing them to contestation (Braun, Moore et al. 2010: 513).

To analyze the practices by which established power relations have been stabilized in the government of animal biotechnology we develop the concept of ‘the moral economy’. Our use of this term is different to its use elsewhere in social studies of science. Drawing on the work of E.P. Thompson (1993), for whom the moral economy, grounded in the moral community, operated as an alternative to the capitalist market economy, studies have examined how moral economies operate within scientific practice, providing ‘systems of primarily non-economic exchange within which scientists seek to balance their personal values—the ideals of their discipline or socio-cultural group—against material and social realities largely generated by macro-level decisions of politics and policy’ (Atkinson-Grosjean & Fairley, 2009: 164; see also Kohler, 1999; McCray, 2000; Rasmussen, 2004). Elsewhere Daston has presented an account that focuses on the affective dimension of the moral economy of science (Daston, 1995). As those who have studied the operation of moral economies within science have noted, they can provide ‘provisional maps for navigating the messy, contingent spaces where societal and scientific values are negotiated’ (Atkinson-Grosjean & Fairley, 2009: 148). However, the focus in such work has been on explicating the negotiation of values *within* the scientific community through a system of exchange based on a common morality. Here, we study the governance community and look at how it is working to institute a system of exchange of moral values.

We draw on recent work in which the moral economy has been conceptualized as a sphere of activity in which values are traded between nation states, as a means of meeting ‘the political need to reconcile the promise of new health technologies with the cultural costs of scientific advance’ (Salter & Salter, 2007: 555). Here, we focus attention on the government of the moral economy of science within the nation state: how is this domain defined, regulated and managed, the diverse value positions circulating within the jurisdiction of the state configured as legal or illegal tender, the exchange rate for trading different values set, and the value preferences of consumers shaped so that satisfaction of their preferences by the individual also enhances the prosperity of the state? Our account is a case study of how these questions have been approached in the UK during the last two decades in relation to the governance of scientific work that mixes animal and human biological material, specifically the examples of genetically modified animals and chimeric embryos.

Genetic modification involves the introduction of human genes into animals, a practice that is carried out for research into human disease (e.g. putting human cancer genes into mice in order to study the development of the disease) and, potentially, therapy (producing human proteins for therapeutic use from human genes inserted into animals). Chimeric, or interspecies, embryos are embryos produced by taking the nucleus (containing the genetic material) from a human cell and introducing it into an animal oocyte from which the nuclear material has been removed. The production of such embryos was proposed by scientists as an alternative to the use of human embryos as a source of stem cell material for research. Genetic modification of animals assumed policy prominence in the early years of this century with the debate having its origin in the controversy surrounding genetically modified crops. The policy discussion of chimeric embryos, which evolved in the latter part of the century's first decade, had its origins in the fierce debates over human embryonic stem cell science, themselves part of a wider history of debate about early human life (assisted reproductive technologies, pre-implantation genetic diagnosis and, most notably, abortion). Significantly, UK policy engagement with the science of GM animals and chimeric embryos took place in the absence of any salient public disquiet, and these debates were selected as being particularly suitable for our study because they allow us to explore the extent to which liberal modes of governance may enable the moral economy to work efficiently in support of science. The empirical material was gathered and analysed as follows. First, consultations were held with UK-based scientific and bioethical experts in the field of animal-human biological mixing to identify the range of public and private organisations that have contributed to the governance debate. These included government committees (eg House of Commons Science and Technology Committee), Research Councils (eg BBSRC), independent regulators (eg Human Fertility and Embryology Authority), advisory non-departmental bodies (eg Animal Procedures Committee) and independent bodies (eg Royal Society). Second, a desk-based survey was conducted of the UK policy and grey literature produced by these organisations. Third, the dominant ethical positions in the policy debate contained in this material were identified and coded in terms of their contribution to our initial theoretical understanding of the operation of the moral economy. As the conceptual framework and organisation of the paper developed, the data was revisited and reframed in an iterative process. Consultations were carried out in the period July 2010 to April 2011; ethical approval for this part of the study was obtained from the King's College London (GGS) Research Ethics Panel. The documentary material studied spanned the period 1998 to 2011. Before presenting the results of this research, we further explicate the role of a moral economy in the governance of science.

## The moral economy: ethics and the liberal government of science

In activities addressing the relationship between science and the public, a model of ‘public understanding’ has given way to one of ‘public engagement’. Approaches adopting the ‘public understanding’ model were premised on a view of the public as lacking requisite understanding of science, such that disputes could be alleviated through education (The Royal Society, 1985: 10). This ‘deficit model’ was much criticised by sociologists of science, who argued that all knowledge is situated, perspectival and contingent (eg Irwin, 1995; Irwin & Wynne, 1996; Jasanoff, 2005). Such claims for the symmetry of knowledge were used to support arguments that ‘lay expertise’ be brought into policy making on an equal footing with the expertise of scientific authorities. The means to resolve tensions, in this case, is considered to be not education of the (deficient) public, but engaging the (expert) public in dialogue with other experts so that a consensus can be achieved.

The ‘dialogic turn’ (Irwin & Michael, 2003: x) in public understanding of science resonates positively with the wider ‘deliberative turn’ in democratic modes of governance. It is thus unsurprising that engagement of the public in dialogue over science has been readily taken up by governments as a necessary component of the policy process designed to ensure the legitimacy of the policy outputs. Initiatives such as consensus conferences, citizens' juries, and public debates such as the *GM Nation?* debate have become a recognised part of the policy landscape. Some form of public engagement exercise has become *de rigueur* for bodies producing reports on scientific developments intended to feed into policy-making.

Such initiatives speak to the liberal valorisation of individual freedom. Government, from the classic liberal perspective, is necessary to ensure individual freedoms, but should be limited to prevent it infringing on those freedoms. It is here that the approach to the analysis of liberal government introduced by Foucault and developed by others may provide a fruitful perspective. Liberalism, in this analysis, is ‘an art of governing that arises as a critique of excessive government’ (Rose, O'Malley, & Valverde, 2006: 84). As such, this art of governing entails a reflexivity on the part of the state, a constant questioning of the proper limits of its activity. This is not a striving to minimise government *per se*, but a concern that the state might be doing too much of the governing (Dean, 2002: 41), encroaching into the domains of governance that exist outside the apparatuses of the state and operate according to their own heterogeneous systems of regulation.

The classic example of such an independent regulatory system is ‘the economy’. In Foucault's analysis, liberal government takes shape with the emergence in political thought of ‘the economy’, a domain functioning according to fundamental natural laws, as an autonomous sphere of rationality working outside the juridical domain of the state (Gordon, 1991: 11). While operating autonomously from the state, the state, in classical liberal thought, retains a vital role in ensuring that this domain of the economy can operate freely: its role is ‘to create regulations that enable natural regulations to work’ (Foucault, 2007: 353). More recent neo-liberal political thought conceives of a more intimate role for the state in the government of the economy. In Foucault's understanding, the key difference between classic and neo-liberal rationalities of government is that while the former sees the market as a ‘quasi-natural reality’ that delimits a sphere in which government cannot act, for the latter the market is only maintained by the actions of government (Gordon, 1991: 41).

In classical liberal thought, the ‘invisible hand’ of the market would work to ensure that the wellbeing of the collective was increased through the actions of individuals seeking to optimise their own wellbeing. In neo-liberal thought, proper government is required to ensure the co-development of individual and collective wellbeing. Government, then, acts in the name of individual and national prosperity (Rose, 1996: 37), and it is through ensuring national prosperity that the security of the state is guaranteed. According to this view, liberal government is not concerned with the direct legitimation of political power over a territory, the aim of sovereign rule exemplified by Machiavelli's Prince. Instead, it is concerned with ensuring the security (as in prosperity) of its people so that this prosperity is not only the State's *raison d’être* but also the route by which it can ensure its continued existence (Foucault, 2000: 322; Gordon, 1991: 19). Political legitimacy is thus achieved at one stage removed, but the operation of power is present and constant.

A key measure of prosperity for contemporary states is their technological capacity (Barry, 2001). A governance problem emerges when cultural values clash, or potentially clash, with values supportive of technological development, so threatening to derail the smooth progress of the scientific enterprise. How can liberal government make sure that science proceeds unimpeded (hence ensuring the prosperity of the state and its people) without governing too much – without imposing unwarranted restrictions on the right of individuals to hold to their own moral beliefs and hence follow their own conceptions of the good? In what follows, we explore how, in relation to animal engineering, liberal government, exemplified by recent UK policy debates, may be able to create a separate problem space for negotiating the clash between scientific and cultural values, outside the realm of the state, and how, adopting neo-liberal governmental practices, it seeks to govern this problem space to ensure that support of science is maintained. This problem space is the ‘moral economy’ of science.

## Constituting the legitimate elements of the moral economy

If the moral economy is to work efficiently as a sphere of private governance distinct from public governance through state institutions, there has to be an understanding of what value conflicts should be situated in which domain. Government through the moral economy must therefore have the ability to allocate ethical issues accordingly and to be seen as acting legitimately in so doing. So for example, the Animal Procedures Committee report on animal biotechnology examines the ethical issues associated with mixing between species, particularly mixing between human and non-human animal, to create hybrid entities. It notes ‘the main opposition to hybridisation probably comes from those who wish to maintain real boundaries between human and non-human, and who retain a conviction that “kinds” are separate creations, each - as it were - designed to embody a particular beautiful form’. It then goes on to state ‘It is no part of our brief to take sides on so large a metaphysical and ethical dispute’ (APC, 2001: 11).

Positioning the question of whether genetic modification is morally wrong because it infringes a ‘natural’ order (whether that order is one designed by God or by Mother Nature) as ‘not one for public policy’ is a classical liberal approach (Weale, 2001: 418). Contemporary liberal politics holds to the principle that the state should remain neutral on moral matters. Far from marginalising such moral questions, positioning them as matters for individuals to determine rather than the state to pronounce on indicates the seriousness with which they are taken by the liberal project as properly belonging to the autonomous realm of the moral economy.

To position the moral economy as an autonomous realm operating according to its own laws is not to say that the state abdicates responsibility for its operation. On the contrary, there is a requirement for the state to take an active role in ensuring that this domain is free to operate unimpeded (Foucault, 2007: 353). Value positions that challenge this principle must be managed (governed) accordingly. Those holding an anti-vivisectionist stance, for example, are managed in such a way that this perspective is devalued or excluded in debates on the use of animals in science. One strategy adopted for devaluing anti-vivisectionist perspectives is to neutralise them through co-option. For example, the NCoB working group on *Ethics of Research Involving Animals* included a representative of the British Union for the Abolition of Vivisection (BUAV), this representative being heavily outnumbered by representatives from science. An abolitionist perspective was thus given its due place under democratic principles, but the abolitionist perspective was not allowed to gain ground. Such a practice for ‘incorporating and regulating the presence of the threatening Other within’ allows the dominant discourse to ‘manage the demands of marginal groups in ways that incorporate them without disturbing the hegemony of the norms that marginalize them’ (Brown, 2008: 27, 36).

The hegemonic strategy of marginalisation through inclusion is complemented by a parallel strategy to deal with those who challenge the rules of the moral economy itself. Radical perspectives propounded by groups such as the Animal Liberation Front (ALF) are excluded – indeed, exclude themselves – from participation in the moral economy. For dealing with such groups, the state is likely to employ the authoritarian measures that are integral to liberal modes of government (Dean, 1996, 2002; Valverde, 1996). For the state, the ALF has de-legitimised its claims through its tactics of violence, so making itself a terrorist operation in the eyes of the policy community and most of the wider policy network. In its report on *Ethics of Research Involving Animals*, the independent Nuffield Council on Bioethics working group agreed with the state that ‘use of violence and intimidation against members of the research community, research institutions, their business partners, family and neighbours, or against organisations and individuals representing animal welfare groups, is morally wrong and politically insidious’ (NCoB, 2005: 264). By using tactics that are morally and legally wrong, the ALF places itself outside the moral economy and, in terms of a liberal democratic approach, excludes itself from the right to participate in the practices by which societal consensus is established according to democratic principles.

In the government of the moral economy, authoritarian measures are not only brought to bear to exclude those interest groups or individuals that ‘disrupt or simply get in the way of the establishment and maintenance of a liberal legal and political order’ (Dean, 2002: 40) – those who will not rather than cannot use reason. They are also applied to the moral arguments that might be enrolled, defining which are legitimate for use in the moral economy, and which are not permitted a place. In its structuring of the moral economy, the problem for the state is not the rational capacity of individual citizens (or the lack thereof), but the definition of the proper and improper use of specific types of rationality in specific arenas of decision-making. As the policy discussion around animal engineering illustrates, the state is at pains to set the standard for the relationship between the formal rationality of the scientific domain and the operation of the moral economy:

We recognise the sincere ethical and moral concerns associated with research of this nature and are therefore concerned that, to respond to these concerns, any regulatory framework associated with use of human-animal chimera or hybrid embryos in research should be transparent and workable. We have, however, been concerned to note that, in certain cases, the serious ethical and moral objections to work of this nature have been clouded through the raising of what appear at first sight to be scientific arguments to support such opposition but which do not stand up to scrutiny. Some of the opposition in responses which we received was based on hostility to science as against Nature. In addition, some throwaway statements concerning the scientific basis for proposed areas of research not only lack supporting evidence but may perhaps be better termed ‘pseudo-science’. We are of the opinion that ethical and moral concerns should be considered within the context in which they are made, and that inappropriate use of science to justify ethical and moral arguments is unhelpful. Inappropriate use of science should be identified and disregarded by Government and other policy-makers. (House of Commons Science and Technology Committee, 2007: 26–27)

Establishing a linkage between the scientific and the moral domains so that the latter becomes dependent on the rational standards of the former is used as a basis for defining the boundaries of the state-sponsored moral economy. Given that scientists and analytically-trained philosophers tend to dominate the working groups and committees producing policy-relevant material to inform debate in the moral economy of science, it is not surprising to find policy documents in which the only valid moral perspective is construed as the one that employs a narrow, technical or formal, rationality. For example, in the BBSRC's *Ethics, Morality and Animal Biotechnology* we find the view that ‘ethical judgements may be argued for and shown to be more or less rational and informed’ and that while consideration of moral concerns should be taken seriously, this is in order ‘to raise the level of the debate and encourage judgements to be made on a rational and considered basis’ (Straughan, 2000: 7). This is an articulation of the perspective that any controversy is a problem of insufficient information and poor reasoning: as Straughan has put it elsewhere, the arguments that genetic modification is unnatural because it crosses species boundaries ‘do not have much ethical significance, resting as they do upon unclear language and unsound reasoning’ (Reiss & Straughan, 1996: 64).

The appeal to facts, derived from empirical science, and to reason, the product of rational thought, as ultimate arbiters in moral debate has a privileged position in policy discourses. In this way the state acts to institute the moral economy as a regulatory domain that, once properly established, has the potential to operate according to its own logic, only requiring state support to ensure the proper rules for its operation are maintained. However, the extract from the House of Commons report on hybrid and chimeric embryos quoted above also suggests the emergence of a supplementary governance challenge for the state. While the report is at pains to distinguish between the scientific and the moral domains, the latter is seen as requiring serious attention in its own right. Following recent mutations in liberal modes of government, there is considered to be a proper role for the state in actively ‘tinkering’ with the ongoing operation of the moral economy to ensure that the desired ends of increased prosperity are met. We now turn to examine how the moral economy is made governable.

## Practices of governing the moral economy

In its *Report on Biotechnology* the APC suggests that ‘If people find the “mixing of kinds” objectionable, nobody concerned with public order, or the use of public funds, can disregard that objection merely because it seems, to some, unreasonable’ (APC, 2001: 19). For contemporary liberal rationalities of government, such attention to a governable space can be seen as bi-directional: moral arguments are regarded as deserving of consideration by the political institutions of the state in the formation of science policy, and this domain of the moral economy is requiring of government by the state in order to ensure it can operate effectively.

Central to the government of this space is the shaping of the ethical preferences of the ‘consumer’ in the moral economy. Engaging in dialogue with the public is viewed as a necessary part of good government in the formulation of policies on areas of emerging science. In the case of animal engineering, the AEBC underpinned its report on animal biotechnology with specially commissioned research into public attitudes (Macnaghten, 2001); the HFEA initiated a public consultation on public attitudes towards hybrid embryos in 2007 (HFEA, 2007); the House of Commons Science and Technology Committee drew on this research in its report on regulation of such embryos, and in addition held its own public seminar, noting ‘This is the first occasion upon which we have held such an event and we found it to be of great value in our deliberations’ (House of Commons Science and Technology Committee, 2007: 7). The AMS work on ‘animals containing human material’ includes ‘a significant programme of public dialogue’ (AMS, 2009a). But this dialogue must, from the perspective of contemporary liberal rationalities of rule, be so governed that the outcomes demanded of good government – increased prosperity realised through technological development – are assured. If, as a proponent of New Labour's policy of ‘personalisation’ has observed, ‘a state that is committed to protecting private freedom must also continuously shape how people use their freedom in the name of the wider public good’ (Leadbeater, 2004: 90), the modes of governance to achieve this happy condition are clearly central to the liberal project.

One stance found in policy debates on genetic modification of animals is that of recognising that the public hold varying value positions, and treating these as analogous to a consumer preference. In the case of food products derived from GM animals, the recommendation was that ‘arrangement should be made to maintain consumer choice about whether to purchase meat or other products from GM and cloned animals’ (AEBC, 2002: 38). In other words, individuals should be able to act to satisfy their value preferences, in this case literally using the (super)market to do so. However, the issues in animal biotechnology range much further than GM food products, with GM animals being used, or potentially used, in a wide variety of ways. In these debates on genetic modification of animals, policy discussions correspondingly considered that attending to public concerns was a more complex matter. Considerable attention is paid in these discussions to research investigating the varied value positions held by the public, establishing that diverse, and often conflicting, moral stances exist. However, the difficult governance question is how to move from a knowledge of the diverse values circulating in the moral economy to the mechanism by which an agreement can be reached on which value positions should form the basis for public policy. There is no ‘invisible hand’ at work in the moral economy. Policy demand and value supply do not achieve a natural equilibrium. A plethora of new techniques for facilitating deliberative dialogue have been trialled: consensus conferences, citizens' juries, public debates, expert-led workshops and so on. These have, however, proved ineffective at producing a compromise agreement and certainly have not achieved consensus. The new tools of deliberative democracy have proved singularly unsuccessful in creating an ideal speech situation in which a communicative rationality can flourish. Instead, as with the *GM Nation?* debate, interest groups with polarised positions have dominated despite avoidance of this phenomenon being an explicit aim of the exercise (Irwin, 2006: 311).

If these new tools of deliberative democracy have proved inadequate to the governance task posed by the moral economy, then other means must be sought. Given that expertise is integral to liberal modes of government, being vital to the ‘government at a distance’ that is its key characteristic (Miller & Rose, 1990; Rose, 1993; Rose & Miller, 1992), the use of expertise as a means for filtering competing value positions is a natural governance path to explore. In the government of values in bioscience and biomedicine, bioethical expertise, in the form of bioethics commissions, committees and the like, has come to have a central role (Salter, 2007; Salter & Salter, 2007). The role of this ‘public bioethics’ (Kelly, 2003) is not to pronounce judgment on the moral acceptability of new science. For example, the Nuffield Council on Bioethics works ‘to identify and define ethical questions … with a view to promoting public understanding and discussion’ (NCoB, 2000: 5). While eschewing a normative role, bioethical expertise has a governmental role. It purifies the ethical discourses existent in society, reframing them in ‘proper’ language and addressing them using ‘proper’ techniques of evaluation (Moore, 2010a, 2010b). Notably, it reconfigures value disputes in ways that are amenable to formally rational debate and decision making (Evans, 2002). This does not mean that such bioethics ignores emotional responses to scientific developments among the public. Rather, it works to distinguish between public opinions with ‘latent ethical potential’ (Moore, 2010a, 2010b: 207), which can be developed into formally rational arguments that can engage with the moral economy and those founded on prejudice which are invalid currency and cannot.

When competing values are in circulation, then, the role of bioethical expertise in the government of the moral economy is as interpreter. But where there is a potential ethical issue but not yet open controversy a further strategy is required to shape the emerging value preferences of consumers. In the case of GM crops a classical liberal approach saw resolution as a matter of individual consumers satisfying their (pre-existing) value preferences through the market. However, in the area of animal engineering, there is an absence of any pre-existing ethical ‘controversy’; as the AMS notes, there is an ‘apparent gulf between current and future scientific practices, and public awareness’ in this area (AMS, 2009b: 3). The task of governing is not to mediate between different deeply held beliefs, trying to find a resolution to the controversy that is acceptable to all. It is instead to pre-empt controversy by identifying and dealing with potential ethical tensions. The state's approach to this practice of anticipatory governance is a neo-liberal one characterised by the active attempt to shape the moral economy through the promotion of certain value preferences over others.

This shaping of value preferences is accomplished through the judicious provision of information. In the public engagement exercises undertaken on the topic of animal engineering, ensuring the public have information not only on the science and its uses but also on the ethical import of that science has been considered vital. For example, as a basis for its consultation on inter-species embryos, the HFEA produced a document ‘which explained some of the social and ethical arguments for and against the research’ (HFEA, 2007: 4.5). The AMS, in its call for a contractor to carry out the public engagement element of its project on animals containing human material, considered that it would be necessary for the contractor to provide participants with information such as ‘what scientific knowledge, and medical benefits, have resulted from such research; how animals containing human material might be used in future research, and what knowledge or medical benefits are anticipated’ (AMS, 2009b: 8). One could interpret this emphasis on providing information as a return to a deficit model, with education a necessary precondition for rational decision-making by the public. However, the consultations set out to do rather more than provide information, in terms of ensuring that those taking part in discussion have the scientific literacy to understand what is involved in animal engineering. They also stress the importance of setting out the arguments for and against the science, as a prerequisite for ‘meaningful engagement’.

In this, the provision of information can be seen as part of a strategy for ‘mobilising the consumer’ (Miller & Rose, 1997) in the moral economy. Miller and Rose examined how the subject of consumption of consumer goods has been assembled by matching the desires of the individual with the outputs of the productive machine, in part by bringing those desires into being through the work of charting them (Miller & Rose, 1997: 31). In the moral economy, governmental practices seek to match the ethical values of the individual with the outputs of the scientific machine, in part by bringing those value positions into being through the various techniques of public engagement with ethical issues in science.

Public engagement initiatives do not use tactics as crude as listing the potential ethical positions and enticing participants to choose from them. Rather, they identify areas of potential ethical tension that should be investigated. For example, the AMS indicated to those tendering for its public dialogue programme that areas to be explored might include ‘where are particular sensitivities (e.g. around particular tissues - reproductive, or neural tissue; or species e.g. primates, domestic animals)’ (AMS, 2009b: 9). In this way the debate is pre-framed in terms that have been set out by bioethical expertise which has highlighted these elements (mixing of brains, mixing between human and non-human primate, use of human embryonic stem cell derived material in animals) as of particular sensitivity (Greely, 2003; Greene et al., 2005). Bioethical expertise is the authority that defines the debate, and hence policy, agenda.

With the debate defined by bioethical expertise, another form of expertise, that of science, comes back into play in shaping the way it develops. In public engagement activities, there is a leading role for scientists in determining what information the public is given about the science. For example, the AMS emphasises that in its public dialogue exercise, ‘The questions involved are to be refined by the Contractor in close consultation with the Working Group’ (AMS, 2009b: 9) which is largely (though not exclusively) scientists. The HFEA exercise included ‘deliberative work’ in which ‘expert speakers were used to illustrate the different issues and arguments relating to the consultation, thereby stimulating questions and debate’ the aim being to ‘explore how the views and opinions of participants changed when exposed to different information’ (HFEA, 2007: 4.9). The provision of information by experts speaking to existing value positions is used to steer the value positions developed in members of the lay public.

Expertise plays a central role in acting on individual subjects to bring into being new ethical preferences, so preparing both ‘the market for the product and the product for the market’ (Thorpe & Gregory, 2010: 273). It is employed as a strategy for developing individual's ethical views in such a way that they are in support of the science. The consultation exercises may be at pains to be neutral and even-handed in presenting information, offering arguments both for and against the science. But by invoking ‘health’ as an outcome of the science, they are tapping into the pre-existing moral discourse in which health is a meta-value the invoking of which legitimates other discourses and practices (Greco, 2004). Consultation exercises may list an equal number of pros and cons, but each point does not carry equal weight in the moral economy: an argument for the science justified in terms of health improvement is likely to outweigh several arguments against the science. Whether this strategy is successful, or whether individuals successfully resist the shaping of their ethical preferences in this fashion, is a matter for further empirical research. However, by suggesting that the science will satisfy the consumer preference for health (including setting out both the medical benefits that have arisen and those that are anticipated (AMS, 2009b: 8)) the bringing into being of value positions supporting the science is facilitated while the articulation of value positions that speak against the science is deterred.

## Conclusion

The way the UK debate over interspecies embryos was managed at the level of policy agenda setting illustrates the success of liberal arts of government of the moral economy of animal engineering. The proposals by scientists to introduce human nuclear material into enucleated animal oocytes to create stem cells for research did not ignite pre-existing moral debates – other than to draw in those opposed to any form of embryo research. Indeed, the main opposition to the research came from the Government of the day, which proposed fairly restrictive legislation, in part on the assumption that the public would be opposed to such technology, an assumption that one observer described as being based on ‘the findings of a flawed public consultation dominated by self-selecting opponents of embryo research’ (Henderson, in Watts, 2009: 17). However, elsewhere in the machinery of the state, the stance was very much in favour of allowing the creation of interspecies embryos (House of Commons Science and Technology Committee, 2007; Joint Committee on the Human Tissue and Embryos (Draft) Bill, 2007). With scientists proactive in promoting the potential benefits of the research in terms of human health (Watts, 2009), and the media on board (Williams et al., 2009), the value positions of the public could be brought into alignment with the demands of government. The consultation by the Human Fertilisation and Embryology Authority concluded that the majority of the public wanted to understand what was proposed and why scientists wanted to do it, and these people tended to shift from an instinctive ‘yuck’ response to acceptance or support of creating chimeric embryos (HFEA, 2007).

We should not neglect other facets of the government of interspecies embryos that pre-dated and preceded its government through the moral economy. It was only a chance remark by a scientist in a press conference that drew media attention to the possibility of this work being undertaken in UK research labs (Fox, in Watts, 2009: 22). Without this inadvertent intervention, it is likely that the production of human/animal chimeric embryos would have been governed as a technical matter, within the existing policies and practices pertaining to the research. When the debate did emerge into the public domain, it was circumscribed by the authoritarian measures integral to liberal government. Thus, when the HFEA consultation received a ‘large number’ of responses from those who were opposed to any type of embryo research, its approach was to ‘distinguish those objecting to the fundamental notion of using human embryos in research, from other respondents, to explore where others might impose limits’ (HFEA, 2007: 5.2), excluding the former from consideration.

In the matter of animal engineering, we have shown how the debate is structured by bioethical expertise, regulating the currency of the moral economy, and how the way the debate plays out is shaped by combined bioethical and scientific expertise, ensuring that the preferences of consumers in the moral economy are such that the satisfaction of these preferences by individuals also satisfies the aims of government. It is through the combination of these various elements of liberal government that the desires of the populace are brought into alignment with the will of the state. This is not an imposition by the state of its will on a populace against their interests for government is a form of pastoral power, a government of all and of each (Foucault, 2000). From the perspective of policy makers, by employing these practices of government, the wellbeing of the individual is enhanced as the security of the state is increased – animal engineering is configured as a technology that can improve both individual and population health, the knowledge generated is understood as both an individual and a social good, and the economic benefits are presented as accruing both to individual citizens and to the national economy in the form of increased competitiveness. Nor is this government the meticulously ordered implementation of a centrally planned programme. The various activities of governing have coherence within the context of a particular governmental rationality – a governmental rationality being the more or less coherent way of thinking about how government should be practised, on what or whom, by whom, and to what ends (Gordon, 1991:3) – but they are not reducible to that rationality.

Analysing the government of animal engineering as a liberal practice allows for a more nuanced understanding than that developed in much social science analysis of science governance. Social scientists have been disappointed with recent initiatives to develop science policy making so that it engages with the wider public, complaining that they have failed to be fully inclusive, engaging only a limited number of viewpoints, in a way that undermines any claims that the consensus reached is properly democratic (Horlick-Jones et al., 2006; Irwin, 2001, 2006; Rowe et al., 2005). Our analysis suggests this is not the failure of a democratic politics but the success of a liberal mentality of rule. Liberalism has demonstrated again its flexibility in adapting to meet new governance challenges, in this case the challenge of public opposition to science. Science may be too important to be left to the scientists, but it is also too important to be left to the public. For the state, the bringing into being of the moral economy and the development of techniques for governing that economy is proving effective in ensuring that its aspirations for enhancing individual and collective prosperity through enhanced technological capacity are assured.
